# Mesenchymal Stem Cells Engineered by Nonviral Vectors: A Powerful Tool in Cancer Gene Therapy

**DOI:** 10.3390/pharmaceutics13060913

**Published:** 2021-06-21

**Authors:** Yuan Ding, Chenyang Wang, Zhongquan Sun, Yingsheng Wu, Wanlu You, Zhengwei Mao, Weilin Wang

**Affiliations:** 1 Department of Hepatobiliary and Pancreatic Surgery, The Second Affiliated Hospital, School of Medicine, Zhejiang University, Hangzhou 310009, China; dingyuan@zju.edu.cn (Y.D.); 11618384@zju.edu.cn (C.W.); sunzq@zju.edu.cn (Z.S.); drwuys@hotmail.com (Y.W.); ywl@zju.edu.cn (W.Y.); 2 Key Laboratory, Precision Diagnosis and Treatment for Hepatobiliary and Pancreatic Tumor of Zhejiang Province, Hangzhou 310009, China; 3 Research Center, Diagnosis and Treatment Technology for Hepatocellular Carcinoma of Zhejiang Province, Hangzhou 310009, China; 4 Clinical Medicine Innovation Center, Precision Diagnosis and Treatment for Hepatobiliary and Pancreatic Disease, Zhejiang University, Hangzhou 310009, China; 5 Clinical Research Center of Hepatobiliary and Pancreatic Diseases of Zhejiang Province, Hangzhou 310009, China; 6 Cancer Center, Zhejiang University, Hangzhou 310009, China; 7 MOE Key Laboratory, Macromolecular Synthesis and Functionalization, Department of Polymer Science and Engineering, Zhejiang University, Hangzhou 310027, China

**Keywords:** mesenchymal stem cells, nonviral vectors, cancer, gene therapy, nanomedicine

## Abstract

Due to their “tumor homing” and “immune privilege” characteristics, the use of mesenchymal stem cells (MSCs) has been proposed as a novel tool against cancer. MSCs are genetically engineered in vitro and then utilized to deliver tumoricidal agents, including prodrugs and bioactive molecules, to tumors. The genetic modification of MSCs can be achieved by various vectors, and in most cases viral vectors are used; however, viruses may be associated with carcinogenesis and immunogenicity, restricting their clinical translational potential. As such, nonviral vectors have emerged as a potential solution to address these limitations and have gradually attracted increasing attention. In this review, we briefly revisit the current knowledge about MSC-based cancer gene therapy. Then, we summarize the advantages and challenges of nonviral vectors for MSC transfection. Finally, we discuss recent advances in the development of new nonviral vectors, which have provided promising strategies to overcome obstacles in the gene modulation of MSCs.

## 1. Introduction

Despite great advances in diagnosis and therapy, cancer is still a leading cause of death around the world [1]. Conventional therapeutic strategies are accompanied by certain unwanted adverse effects due to a lack of specific targeting; thus, innovative therapeutic approaches are urgently needed.

Since the discovery of the tumor homing properties and immune privileged status of mesenchymal stem cells (MSCs), they have been considered promising tools for anticancer treatments [2,3]. MSCs can be converted into cellular vehicles and deliver tumoricidal agents to tumors in a targeted manner. Two kinds of methods can be employed to achieve this goal. The first method is gene-directed enzyme prodrug therapy (GDEPT), in which genetically engineered MSCs can produce specific enzymes that convert nontoxic prodrugs into active cytotoxic derivatives [4]. The second strategy is the targeted delivery and local production of bioactive molecules (cytokines, chemokines and ligands) in tumor sites via genetically modified MSCs [5].

Currently, there are more than 50 clinical trials investigating MSC-based cancer gene therapy that are listed on the clinical trial database of the National Institutes of Health, indicating the thriving progress in this field. However, some questions need to be addressed, such as the long-term safety and effectiveness, which are largely determined by the selection of candidate genes and delivery vectors. In this review, we focus on gene delivery vectors that have been used for MSC-based cancer gene therapy. The majority of studies have exploited viral vectors for transfection of MSCs [6]. Although viral gene delivery offers a higher transduction efficiency, several drawbacks associated with viruses, such as carcinogenesis and immunogenicity, have drawn attention from both researchers and administrators [7,8]. Nonviral vectors have the potential to overcome these limitations because they have better biocompatibility and induce a relatively low level of immune response [9,10]; however, classical nonviral vectors can exhibit disappointing transfection efficiency [11,12].

Due to developments in the fields of material sciences and nanotechnology, the low efficiency of nonviral vectors in MSC transfection may be overcome. Studies have combined classical vectors into new hybrid vectors or yielded new artificial materials as delivery vectors, which can greatly improve the transfection efficiency without a significant loss of cell viability [10,13]. In addition, some studies have developed functional nonviral vectors, which have broad application prospects in MSC-based cancer gene therapy. It is anticipated that new strategies involving nonviral vectors will lead to a new era of MSC-based cancer gene therapy.

In this review, we first summarize recent advances in understanding the relationship between MSCs and tumors and discuss the advantages and potential limitations regarding the utilization of MSCs as anticancer tools. Then, we review the current knowledge about MSC-based cancer gene therapy. Moreover, we briefly comment on the main advantages and disadvantages of viral and nonviral vectors and point out the potential benefits and challenges of using nonviral vectors to engineer MSCs. Next, we review the different types of nonviral vectors. Finally, we highlight recent advances in MSCs engineered using nonviral vectors, representing promising strategies to address the current challenges in gene delivery of nonviral vectors.

## 2. The Pros and Cons of MSCs for Tumor Therapy

### 2.1. Definition of MSCs

MSCs are nonhematopoietic stem cells with high differentiation potential and self-renewal abilities [14]. They can differentiate into various cell types, such as adipocytes, osteoblasts and chondrocytes [15]. To date, they have been found to exist in almost all tissues [16] and can be easily isolated from bone marrow, adipose tissue and umbilical blood [17]. MSCs can be highly heterogeneous; hence, most studies define MSCs according to the minimal criteria proposed by the International Society for Cellular Therapy (ISCT). The criteria include the following: MSCs must be plastic-adherent in standard culture conditions; MSCs must express CD105, CD73 and CD90 and lack expression of CD45, CD34, CD14 or CD11b, CD79α or CD19 and HLA-DR surface molecules; MSCs must have the ability to differentiate into osteoblasts, adipocytes and chondroblasts under suitable conditions in vitro [18]. Additionally, CD146 is also considered to be a reliable marker of MSCs [19]. However, it is worth mentioning that MSCs from different tissues exhibit different biological activity and markers. A study indicated that MSCs were phenotypically heterogeneous and showed diverse differentiation potential and secretion of bioactive factors associated with tissue origin [20]. Therefore, selecting MSCs from different tissues as anticancer tools is also likely to have an impact on the efficacy.

### 2.2. The Advantages of MSCs for Tumor Therapy

#### 2.2.1. Tumor Homing Properties

MSCs can specifically migrate to wounds and actively participate in the wound healing process [21]. Tumors, regarded as “wounds that never heal” [22], have been proven to possess an ability to recruit MSCs. The phenomenon of MSCs homing to tumors has been observed in various cancers, such as breast carcinomas, glioma, pancreatic cancer and hepatocellular carcinoma [23,24,25,26]. Although the exact mechanisms are still unclear, studies have found several factors responsible for the tumor tropism of MSCs. Tumor cells and other components in the tumor microenvironment (TME) can secrete various soluble factors, such as CC chemokine ligand 2 (CCL2), CCL5, CXC chemokine ligand 12 (CXCL12), transforming growth factor β (TGF-β) and interleukin 6 (IL-6) [27,28,29,30], which can regulate MSC chemotaxis and favor the homing of MSCs to the tumor.

#### 2.2.2. Immune Privileged Status

The low immunogenicity of MSCs is associated with the low expression level of MHC class I and lack of expression of MHC class II or costimulatory molecules (B7-1, B7-2 or CD40) [31,32]. In addition, MSCs possess great immunomodulatory potential. MSCs can produce various factors, such as inducible nitric oxide synthase (iNOS), CXCL9 and TGF-β, which suppress the immune response by inhibiting B and T cell proliferation and promoting the generation of regulatory T cells [33,34]. Additionally, MSCs can exert immunomodulatory functions via interactions with immune cells through cell-to-cell contact. For example, human placenta MSCs express high levels of the cell adhesion molecules programmed death ligand 1 (PD-L1) and PD-L2, which can inhibit T cell proliferation and suppress CD4^+^ T cell activation [35]; therefore, MSCs hardly activate the host immune response even without HLA matching [36], which also protects MSCs from immune detection and immune clearance [37].

Moreover, preconditioning MSCs have more recently been shown to increase their immunomodulatory potential [38]. Hypoxia-preconditioning or preconditioning by priming with immunomodulatory factors (IFN-γ, TNF-α) can further enhance the ability of MSCs to inhibit T cell proliferation [39,40]. It reminds us that it is feasible to pretreat MSCs to increase or decrease their immunomodulatory functions.

Owing to the properties of MSCs, they are ideal cellular vehicles for the delivery of anticancer agents, providing improved bioavailability compared to conventional approaches.

### 2.3. The Limitations of MSCs for Tumor Therapy

#### 2.3.1. Protumorigenic Roles

**Figure 1:**
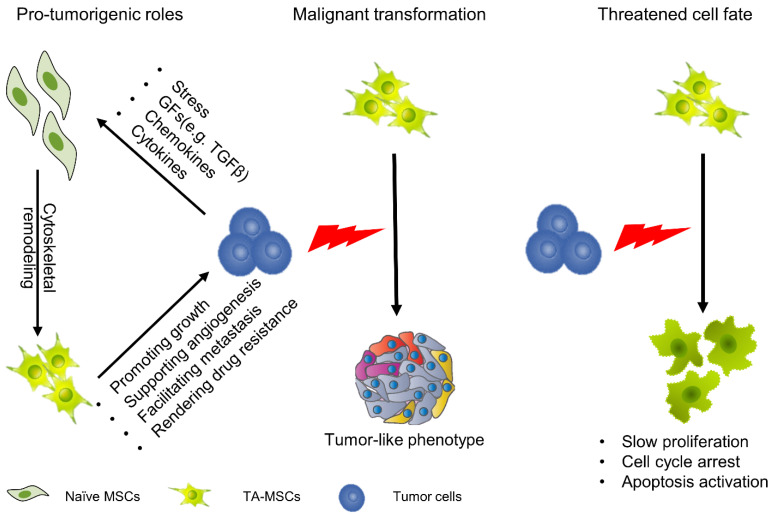
Cellular function and fate of MSCs in the TME. MSCs can migrate to tumors, and under the education of tumor cells, evolve into TA-MSCs. In this process, the function and fate of MSCs undergo profound changes. TA-MSCs play an important role in promoting tumor growth, supporting tumor angiogenesis, facilitating tumor metastasis and rendering tumor drug resistance. Meanwhile, some studies have found that TA-MSCs undergo malignant transformation. On the other hand, some studies have demonstrated that TA-MSCs show significant reductions in cell viability with cell cycle arrest.

We must clearly recognize that MSCs are not simple delivery vehicles but cells with active physiological processes. Although there are considerable discrepancies in the impacts of MSCs on tumor progression, most studies tend to believe that MSCs play active roles in tumor initiation and progression [41]. Numerous studies have explained the function of MSCs in tumors from different perspectives. They have revealed that MSCs play important roles in promoting tumor growth, supporting tumor angiogenesis, facilitating tumor metastasis and rendering tumor drug resistance (Figure 1) [42,43,44,45]. In this regard, when we apply MSCs as anticancer vehicles, they might also provide prosurvival signals for tumor cells, which may result in tumor progression; thus, we need to strictly evaluate the protumorigenesis and antitumorigenesis effects of genetically engineered MSCs. Moreover, another direction for development is to increase the efficiency of target gene expression to reduce the number of MSCs.

#### 2.3.2. Cellular Fate of MSCs in the TME

When MSCs migrate to tumor sites and continuously remodel the tumor niche they undergo profound changes; however, there are two strikingly different views. On the one hand, some studies have demonstrated that MSCs undergo malignant transformation with exposure to tumor cells. In a glioma model, tumor-derived exosomes or cytokines induced a tumor-like phenotype in MSCs with enhanced capacity for proliferation and migration [46,47]. On the other hand, studies have also found that the fate of MSCs is seriously threatened after exposure to TME. In a hepatocellular carcinoma model, MSCs show a significant reduction in cell viability with cell cycle arrest (Figure 1) [48,49]. In either case, these properties are not conducive to MSC-based cancer therapy because the former is related to safety issues and the latter is related to efficacy issues.

Although MSCs still have some defects, these do not preclude MSCs from becoming an ideal anticancer tool. More comprehensive knowledge of MSC physiology within the TME and the innovative genetic modification of MSCs might improve the proposed therapy. We always believe that MSCs could have bright prospects in cancer therapies, especially in advanced malignancies.

## 3. MSC-Based Cancer Gene Therapy

**Figure 2:**
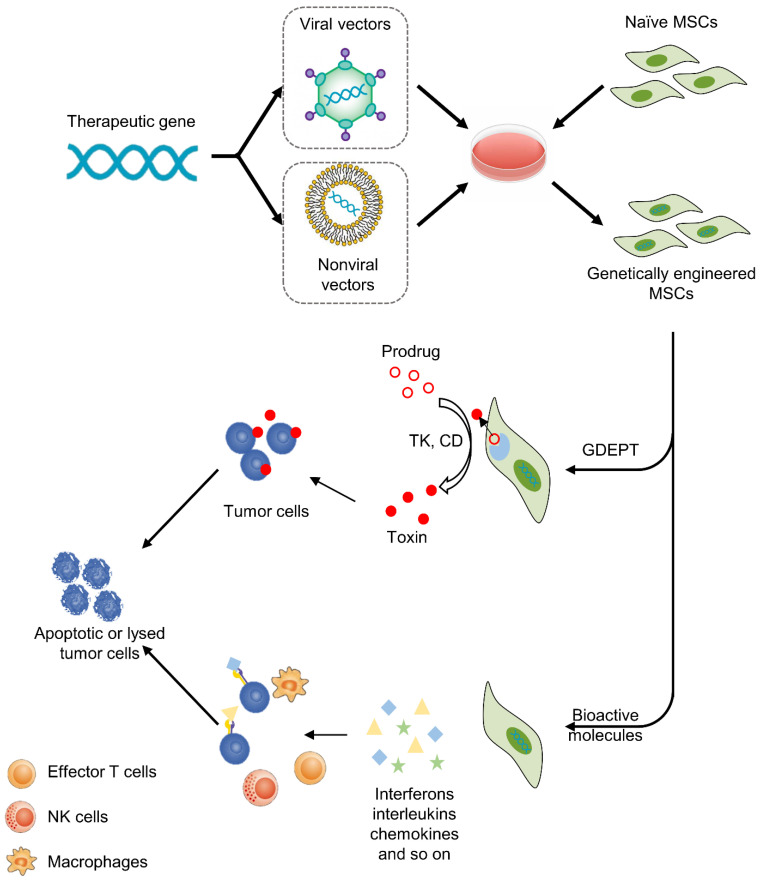
MSC-based cancer gene therapy. Therapeutic genes are transferred into MSCs by viral or nonviral vectors in vitro, then MSCs deliver therapeutics to tumors. There are two basic strategies, including GDEPT and therapy with bioactive molecules. For GDEPT, MSCs can produce enzymes that can convert prodrugs into activated cytotoxic drugs and induce apoptosis and necrosis of cancer cells. As another strategy, MSCs can secret bioactive molecules, which not only exert direct antitumor effects but also activate immune cells and enhance the anticancer immune response.

Gene therapy is a promising and challenging approach in the treatment of cancer. The purpose of gene therapy is to transfect therapeutic genes into target cells to achieve beneficial biological effects [50]; however, the systemic delivery of gene vectors (viral or nonviral) appears to be poorly efficient, at least in part due to a lack of specific targeting. As such, an increasing number of studies have applied MSCs as cellular vehicles to deliver therapeutics to tumors. In this section, we will review the genetic manipulation of different MSCs for anticancer treatments (Figure 2).

### 3.1. Gene-Directed Enzyme Prodrug Therapy (GDEPT)

This approach uses suicide genes to modify MSCs so that MSCs can express specific enzymes that convert nontoxic prodrugs into toxic products [51]. Then, engineered MSCs are administered and targeted toward tumor sites. Subsequently, prodrugs are injected into the body and specifically activated by enzymes secreting MSCs within the TME. In the last stage, activated cytotoxic drugs can induce apoptosis and necrosis of cancer cells. There are two advantages of this anticancer approach. One is the minimization of off-target toxicity, while the other is the amplification of toxicity via the bystander effect. Drugs not only kill tumor cells but also lyse other cellular components in the TME, such as immune cells, and then initiate a cascade of immune responses, leading to more effective cancer death [4]. At present, the most common enzyme-prodrug complexes are herpes simplex virus thymidine kinase complexed (HSV-TK) with ganciclovir (GCV) and yeast cytosine deaminase (CD) or fusion yeast cytosine deaminase::uracil phosphoribosyl transferase gene (CDy::UPRT) with 5-fluorocytosine (5-FC). The former produces GCV triphosphate, which can inhibit DNA polymerase and cause replication failure. The latter converts 5-FC to 5-fluorouracil (5-FU), which has been widely used for cancer chemotherapy. The anticancer effects of this approach have been verified in a variety of tumors, including glioma, melanoma, prostate cancer and gastrointestinal cancer (Table 1). In addition, several clinical trials have been implemented to test the safety and effectiveness of this anticancer strategy. Ten patients with advanced gastrointestinal adenocarcinoma were enrolled in TREAT-ME-1, a phase 1/2 clinical trial. All patients were treated with HSV-TK-MSCs and GCV. The results showed that this therapy was safe and tolerable, with appreciable efficacy [52].

Activated drugs are also highly toxic to MSCs themselves; thus, MSCs will die in the process. As such, most studies have used larges dose of genetically engineered MSCs, with the ratio of MSCs to cancer cells reaching as high as 4:1 (Table 1). This most likely causes unexpected side effects due to the protumorigenic function of MSCs. As a result, bioactive molecule therapy is on the rise.

### 3.2. Therapy with Bioactive Molecules

This approach was first used in 2002. MSCs were modified to express interferon-beta (IFN-β), then the MSCs were administered to tumor-bearing mice and IFN-β was produced locally at tumor sites, which resulted in significant inhibition of malignant cell growth in vivo [65]. Since then, an increasing number of studies have tried to apply different agents for the treatment of cancers. These bioactive molecules include interferons (e.g., IFN-α, IFN-β, IFN-γ), interleukins (e.g., IL-2, IL-7, IL-12, IL-15, IL-25), chemokines (e.g., CX3CL1) and other agents (e.g., TRAIL, TSP-1, NK4) (Table 2). Although different bioactive molecules have their own unique anticancer mechanisms, in general they are mainly achieved by promoting apoptosis of tumor cells or activating the immune response. For example, TRAIL is the ligand for death receptors. TRAIL can bind with its receptors death receptor 4 (DR4) and DR5 to initiate caspase-mediated apoptosis [66]. Given the expression of DR4 and DR5 on the membranes of tumor cells but the absence of DR4 and DR5 on almost all MSCs [67], this therapy is more precise and targeted than other therapies. Another example is IL-12 and IL-15, which not only exert direct antitumor effects but also activate T lymphocytes and NK cells in the TME, enhancing the anticancer immune response [68,69,70].

The greatest advantage of this approach is that it can genetically modify MSCs and preserve their viability; therefore, with this approach, the number of MSCs needed can be reduced. In some studies, only a 1:20 ratio of MSCs to cancer cells could also achieve ideal anticancer effect. Notably, this approach will not have a fatal effect on the viability of MSCs, and MSCs can survive over long terms in the body; therefore, the long-term safety of this approach still requires further evaluation.

### 3.3. Viral or Nonviral Vectors in Cancer Gene Therapy

The overwhelming majority of gene therapy preclinical and clinical trials conducted so far have used modified viral vectors, such as retroviruses, lentiviruses and adenoviruses, to deliver genes. Although the gene transduction efficiency of viral vectors has substantially advanced the field of gene therapy, security issues still need to be considered. Similar to lentiviral vectors and retroviral vectors, viral vectors can integrate into the host genome and induce insertional mutagenesis [85], especially first-generation retroviral vectors. In a clinical study using retrovirus to modify hematopoietic stem cells, it was found that all patients developed acute lymphoblastic leukemia [86,87]. Although viral vectors have been improved in recent years and there is no conclusive evidence to prove their carcinogenicity, immunogenicity and pathogenicity at present, the long-term potential harm is still noteworthy [88,89,90]. Therefore, we must be cautious in applying viral vectors in clinical trials.

Nonviral vectors seem to have great potential to overcome these limitations, particularly with respect to safety. For example, nonviral vectors have lower immunogenicity than viral vectors and they will not be affected by the pre-existing immunity of the hosts [91]. Meanwhile, nonviral vectors allow thousands of DNA copies to be delivered into individual cells, hence increasing the genetic payload for the delivery of therapeutic agents [92]. However, to date, nonviral vectors have rarely been developed clinically because of their low delivery efficiency compared with viral vectors [93]. Owing to developments in material sciences, this drawback may be about to change. In the next part, we will briefly introduce nonviral vectors for gene delivery and then address advances in nonviral vectors for MSC.

## 4. Nonviral Vectors

Presently, two widely used systems for nonviral gene delivery are cationic organic carriers (lipids, polymers and peptides) and inorganic nanoparticles [94]. Here, we will briefly summarize the main advantages and disadvantages of these vectors, as well as their application in MSCs (Table S1).

### 4.1. Cationic Organic Carriers

#### 4.1.1. Lipid-Based Carriers

Lipid-based carriers are the earliest and most widely used for nonviral gene transfection. As early as 1980, Fraley delivered liposome-encapsulated SV40 DNA into cells [95]. Over time, lipid-based derivatization carriers began to appear, such as lipid complexes, liposomes and mono- or multivalent cationic lipids [96].

Liposomes are self-assembling systems. Generally, liposomes are composed of cationic lipids and helper neutral lipids such as cholesterol. They are spherical vesicles composed of phospholipid bilayers and have the ability to encapsulate nucleic acids [97]. When anionically charged DNA molecules are electrostatically complexed with liposomes, liposomal DNA complexes are formed [98]. Liposomes are mainly taken up by endocytosis [99], and these carriers are characterized by numerous benefits, including easy synthesis, biodegradability and safety [13].

Monovalent or multivalent cationic lipids are composed of three principal domains: a hydrophilic head group, a hydrophobic tail and a spacer [91]. The hydrophilic domain is usually composed of one amino group (monovalent, such as N-[1-(2,3-dioleyloxy) propyl]-N,N,N-trimethylammonium chloride (DOTMA) and 1,2-bis(oleoyloxy)-3-(trimethylammonio) propane (DOTAP)) or several amino groups (multivalent, such as 2,3-dioleyloxy-N-[2(sperminecarboxamido) ethyl]-N,N-dimethyl-l propanaminium trifluoroacetate (DOSPA) and di-octadecyl-amido-glycyl-spermine (DOGS)), and is responsible for condensing negatively charged oligonucleotides. Meanwhile, the nature of the hydrophilic domain can dramatically affect the transfection properties of lipid-based carriers [100]. The hydrophobic tail is formed by aliphatic chains, steroids or fluorinated tails, and studies have found that it plays an important role in liposome stabilization, endosomal escape and the toxicity of the vectors [101,102]. Ether, ester, amide or disulfide linkages are usually used as spacers to link the hydrophilic head with the hydrophobic tail [103].

Currently, the representative cationic lipid carriers on the market include DOTMA, DOTAP, DOSPA and DOGS; these carriers are characterized by low toxicity and immunogenicity [104]. Among them, transfection reagents represented by Lipofectamine^®^ have been commercialized and even hailed as the “gold standard” for nonviral gene delivery by many studies [105]. The most widely used Lipofectamine^®^ reagents are Lipofectamine^®^ 2000 and Lipofectamine^®^ 3000, and their ease of use and low toxicity have been confirmed.

#### 4.1.2. Cationic Polymers

Cationic polymers can bind negatively charged nucleic acids through static electricity and then condense them into nanoparticles for gene delivery. Because the physical shape of cationic polymers can significantly influence the gene delivery efficiency, their vast chemical diversity and functional potential have aroused wide interest from researchers. At present, the most common polymers include poly-l-lysine (PLL), polyethyleneimine (PEI), poly(amidoamine) dendrimer (PAMAM) and poly (2-(dimethylamino)-ethyl methacrylate (PDMAEMA).

PLL is a synthetic linear polypeptide containing repeating l-lysine residues and was the first cationic polymer vector used for gene transfection [106]. Because PLL only contains primary amines, it is the safest polymeric transfection vector [107]; ^h^owever, it also affects the transfection efficiency of PLL, as the primary amines are often protonated at physiological pH [108]. To overcome this limitation, some studies have integrated other polymers into PLL. An example is PLL covered with the hydrophilic polymer PEG (polyethylene glycol) to enhance its transfection [109].

PEI is a three-member ring of ethyleneimine, and according to its topology, PEI can be divided into linear PEI (lPEI) and branched PEI (bPEI); bPEI is more widely used because it contains primary, secondary and tertiary amines. Its unique amine groups can give it a stronger buffering capacity with a proton sponge effect [110,111]; however, the primary feature accounting for PEI efficacy is also the source of its high toxicity profile. Studies have confirmed that PEI can induce significant cytotoxicity [112]. One of the main reasons for this result is that the positive surface of PEI will aggregate in a time-dependent manner under physiological conditions [113]. Numerous studies investigating the modification of PEIs are ongoing. For example, PEG was used to cover the positive charge on the surfaces of PEIs [114].

PAMAM is a kind of dendrimer-like, star-branched polymer with a central core [115]. There are numerous primary and tertiary amines on the surface of PAMAM, meaning it can effectively condense and deliver genes [116]. Similar to PEI, PAMAM dendrimers have high cytotoxicity [117]. In addition, the effective release of nucleic acids from PAMAM complexes in the cell is a huge challenge [118].

PDMAEMA is a water soluble polycation [119] and its powerful proton sponge effect gives it satisfactory transfection efficiency. Its nonbiodegradability and biological incompatibility make PDMAEMA quite cytotoxic. In addition, the higher molecular weight of PDMAEMA leads to higher transfection efficiency and cytotoxicity [120]. Approaches to balance the relationship between toxicity and efficiency are worth considering.

Polymeric micelles with a specific core–shell architecture formed via self-assembly of amphiphilic polymers are usually more stable than classical polymers. Moreover, the size of polymeric micelles is smaller, with diameters ranging from 10 to 100 nm, which makes them become internalized more efficiently, making them better candidates for gene delivery [121]. Polymeric micelles currently used for gene delivery usually contain two-part structures—one part is a polycation, such as PEI, PLL or PDMAEMA, while the other part is a hydrophilic polymer including PEG or dextran [122]. To date, siRNA-PEG–PDMAPMA [123], siRNA-PEG–PEI [124] and siRNA-PEG–PLL polyplex micelles [125] have been prepared for gene delivery. Moreover, the stimuli-sensitive breakdown provides them more effective and precise means to deliver genes. The stimuli-sensitive micelles can respond to intracellular (pH, reduction, oxidation and enzyme) or extracellular (temperature, magnetic field and light) stimuli by disassembling their structure and release genes, thereby minimizing off-target effects [126]. For example, some studies have utilized pH-responsive building blocks, such as pHis and poly(β-aminoester), to produce pH-responsive micelles, which undergo pH-triggered charge conversion and resultant alterations of polymeric property for faster genes release and endosomal escape [127,128]. Additionally, other chemical moieties have been incorporated into the micelles to produce smart multifunctional micelles, such as light-sensitive moieties (visible-light-sensitive BODIPY and UV-light-sensitive 2-nitrobenzyl linkers) [129]. Although polymeric micelles have shown numerous possible benefits, their low loading efficiency is still a great challenge.

#### 4.1.3. Peptides

Compared with other nonviral vectors, peptides are generally less toxic. Currently, widely used peptides usually contain basic amino acids such as lysine, arginine or ornithine so that they can condense oligonucleotides effectively [130]. However, the main drawback of peptides is poor transfection efficiency.

The incorporation of functional peptides seems to be a promising strategy. According to biological function, they can be divided into cell-penetrating peptides, targeting peptides, endosome disrupting peptides and nuclear localization signal peptides [131]. These functional peptides enable gene carriers to enter cells more efficiently and enhance gene expression.

Cell-penetrating peptides (CPPs) belong to a class of short peptides that are able to penetrate across cell membranes and have been demonstrated to facilitate the transfection of nonviral gene delivery vectors [131,132,133]. Human immunodeficiency virus (HIV) twin arginine translocation (TAT) peptide is the most commonly used CPP for gene delivery. In our previous study, we synthesized the TAT peptide and conjugated it with cationic metal nanoparticles, and found that TAT can significantly enhance cellular and nuclear entry and improve transfection efficiency for stem cells [134].

In addition, other types of CPPs have shown great potential. For example, antimicrobial peptides (PEPs) can insert into the plasma membrane and have the potential to facilitate the cellular uptake of genes. As such, in our previous study, we used PEP to functionalize cationic gold nanoparticles (AuNPs) and integrated both advantages. AuNPs can effectively condense DNA, and PEP is beneficial to cellular uptake and internalization to achieve high transfection efficiency [135]. Other CPPs, such as low-molecular-weight protamine (LMWP) [136], α-helical cell-penetrating peptide [137] and R9-LK15 [138], have also been proven to improve the transfection of vectors into stem cells.

Targeting peptides can specifically bind to receptors on the cell surface so that the vectors can internalize via receptor-mediated endocytosis. For example, neural ganglioside GD2 is a special surface marker of MSCs; thus, in a previous study, the authors described scAbGD2 (GD2 single chain antibody) attached to PEI and the transfection efficiency was significantly improved [139]. Similarly, other studies have used vascular endothelial growth factor receptor (VEGFR) agonists and antagonists to target VEGFR-1 [140], RVG to target nicotinic acetylcholine receptors [141,142] and arginine-glycine-aspartic acid (RGD) to target integrins [143], which all achieved good results.

In addition to enhancing cell adhesion and endocytosis, there are some targeting vectors that can improve intracellular trafficking. Nuclear targeting vectors play an important role [144]. SV40 DNA targeting sequences (DTSs) are capable of stimulating nuclear entry by binding transcription factors with nuclear localization signals and are beneficial for nuclear import [145].

More importantly, these functional peptides can be combined with each other through covalent bonds or combined with other types of nonviral vectors to form a new hybrid vector. The potential of this change gives peptides broader application prospects.

### 4.2. Inorganic Nanoparticles

Biocompatible inorganic nanoparticles are readily prepared, stable and nontoxic, and have been widely researched. Compared with organic carriers, they are smaller and it is easier to maintain their uniformity [146]. Due to their small size and high surface area to volume ratio, they can easily penetrate cell membranes and demonstrate a high rate and dense loading of genes [147]. Therefore, inorganic nanoparticles have gradually become a powerful tool for gene therapy.

At present, the classification of inorganic nanoparticles mainly depends on the nature of the material, such as the metallic nanoparticles, iron oxides, calcium phosphate and carbon nanotubes. Different types of inorganic nanoparticles have their own advantages.

#### 4.2.1. Metallic Nanoparticles

Among metallic nanoparticles, gold nanoparticles (AuNPs) are the most widely studied. They share most of their attractive qualities with other inorganic nanoparticles. AuNPs can not only effectively condense DNA but also allow the surface to be modified by cationic molecules, such as amino acids and polymers [148]. This provides convenience for the functional reconstruction of AuNPs [149].

Although AuNPs have been more thoroughly tested for the delivery of nucleic acids due to their high stability and antioxidant properties, silver nanoparticles (AgNPs) could be a valuable alternative due to their higher reactivity and lower cost, which could increase the range of possibilities for surface functionalization [150]. For example, AgNPs surface-modified with carbosilane dendrons have showed excellent stability and can be taken up efficiently by cells [151].

Additionally, cerium oxide nanoparticles (CeO_2_) have attracted much attention due to their pH-dependent antioxidant activity. One study found that PEI-associated CeO_2_ can improve the transfection efficacy and decrease the cytotoxicity compared with PEI [152].

#### 4.2.2. Iron Oxides

Iron oxides usually consist of a magnetic core (Fe_3_O_4_) or maghemite (g-Fe_2_O_3_), with magnetic characteristics being their main advantage [153,154]; thus, it is possible to target delivered genes via iron oxides under magnetic guidance [155,156]. Using an external magnetic field, magnetic iron oxide can adhere to the cell membrane within minutes, which will lead to the increased internalization of delivered genes [157]. In addition, magnetite nanoparticles are surface-modified with cationic polymers to promote gene release after endosomal escape and to enhance gene transfection efficiency [158].

Iron oxides can also be used as magnetic resonance imaging (MRI) signal enhancers to track the movement of cells or genes in the body [159]. In a previous study, superparamagnetic iron oxide nanoparticles exhibited high transfection efficiency in delivering genes to MSCs but hardly affected the viability of MSCs [160].

#### 4.2.3. Calcium Phosphate

Calcium phosphate (CaPi) is a component of bones that has excellent biocompatibility; therefore, it is widely applied in bone tissue engineering.

In addition, pH-dependent solubility is another important property of CaPi. It enables CaPi to degrade in acidic tumor microenvironments or endolysosomal organelles [161,162,163] and ensures long-term safety. Recently, nucleic acids have been encapsulated into the CaPi system, and the therapy efficiency has been verified by large numbers of researches. More recently, CaPi was synthesized with a size range of 91 to 6500 nm, then the bispecific antibody gene was loaded into the CaPi system. The results showed that the CaPi minicircle DNA system has high gene delivery efficiency and anticancer immunotherapy efficacy [162]. However, the reproducibility of the CaPi preparation process is poor [164,165].

#### 4.2.4. Carbon Nanotubes

Carbon nanotubes are cylindrical graphene sheets that are characterized by a high length/diameter ratio [166]; therefore, they can be loaded with a large amount of cargo [167]. Due to their attractive properties, consisting of easy translocation across the cell membrane, they were first used for gene delivery [168]. However, there are still several issues that need to be resolved regarding carbon nanotubes, such as their insolubility, nonbiodegradability and low biocompatibility [169].

### 4.3. Other Functional Nonviral Vectors

In addition to the nonviral vectors mentioned above, numerous researchers have created novel hybrid vectors. The purpose of this strategy is to maintain or maximize individual advantages while neutralizing corresponding drawbacks. In this context, some vectors with unique functions have emerged. These functional nonviral vectors not only have the ability to deliver genes into cells but also have other functions, such as fluorescence imaging and ultrasonic or optical control [170]. It is precisely because of this transformation that nonviral vectors have unparalleled broad prospects.

Using fluorescent visible vectors, we can effectively track and monitor the migration behavior and organ-specific accumulation of MSCs in the body. Multifunctional fluorescent carbon dots are representative fluorescent vectors that possess low toxicity and excitation-dependent fluorescence; therefore they hold great promise for bioimaging [171]. In addition, researchers have developed a variety of fluorescent vectors by linking fluorescent compounds to vectors or surface modifications. For example, fluorescent polymers have been synthesized by conjugating PAMAM dendrimers to fluorescein [172]. In addition to organic fluorescent vectors, inorganic fluorescent vectors such as tetranuclear ruthenium (II) [173] and semiconducting polymer dots [174] are powerful tools that have been reported to show stable and continuous fluorescence.

Ultrasound-triggered vectors can be used as another safe and cost-effective strategy for gene therapy. In a previous study, the researchers designed an ultrasound-triggered phase-transition cationic nanodroplet, consisting of a cationic polymer as the core for loading perfluoropentane (PFP) and DNA and a shell for nanoparticle stabilization. The vectors were stable in serum-containing conditions, and with ultrasound irradiation microbubbles were generated, leading to a good US contrast effect. As a consequence, the gene transfection efficiency was significantly enhanced [175].

Photodynamic therapy is also an attractive research field. Researchers have designed vectors and codelivered them with a photosensitizer, ultimately achieving photoprogrammable gene delivery. Near-infrared light can result in reactive oxygen species destroying biofilms by activating photosensitizers [176]. In addition, this strategy can facilitate both the internalization and endosomal escape of gene complexes. It has been proven to enhance transfection efficiency in MSCs, while it only has a negligible effect on the cell viability of MSCs [8,177]_._

## 5. Perspectives

**Figure 3:**
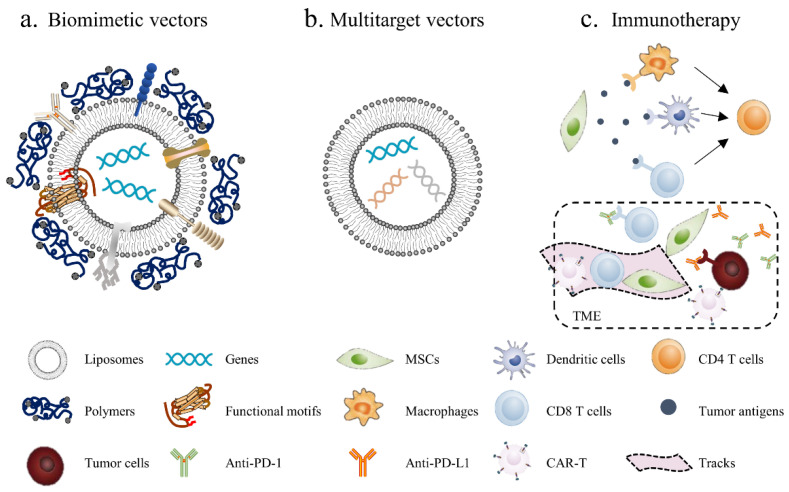
Novel strategies for MSC-based cancer gene therapy: (**a**) biomimetic nonviral vectors are designed to mimic viral characteristics, using liposomes or polymers to construct the scaffolds and then embed certain important functional motifs into them; (**b**) multitarget vectors are used to transfect different anticancer factors through the same vector; (**c**) MSC-based immunotherapy, using MSCs to deliver tumor antigens, activate immune cells and induce local immune responses. Additionally, MSCs can be used to generate “tracks” and guide CAR-T and other immune cells into the tumor site, meanwhile blocking immunosuppressive signals in the TME by secreting PD-1/PD-L1 antibodies.

There is no doubt that MSC-based therapy holds much potential for anticancer treatments; the development of nonviral vectors represents a new era of cancer gene therapy, and the safety of these vectors gives this therapy more potential for clinical transformation. Based on the current research, we believe that therapy with nonviral vectors still has huge room for improvement in the following areas (Figure 3):

1. Biomimetic vectors: The greatest challenge currently is determining how to improve the transfection efficiency of nonviral vectors. This will not only enhance the cellular uptake of genetic materials but also activate endosomal escape and nuclear import. The structure and function of the virus can provide insights. Biomimetic nonviral vectors are designed to mimic viral characteristics and overcome cellular barriers [178]. For example, liposomes or polymers can be used to construct the scaffolds of the vector and then to embed certain important functional motifs into them, such as CPPs, fusion peptides and histone H1. The key for this strategy is to design and arrange their topologies to preserve each domain’s functionality and not to interfere with the other domains’ functions;
2. Multitarget vectors: At present, a vector usually delivers one therapeutic agent; however, nonviral vectors have the potential to deliver larger genetic payloads, meaning different anticancer factors can be transfected through the same vector to exert a synergistic anticancer effect;
3. MSC-based immunotherapy: Tumor immunotherapy has made exciting progress, although it is still not a game-changing solution due to the low cancer immunogenicity in the TME; thus, inducing the immune response within the tumor seems to be an effective solution. Using MSCs to deliver tumor antigens, activate immune cells and induce local immune responses seems to be a promising antitumor strategy. In addition, tumor-associated immunosuppressive cells or signals can be targeted to enhance anticancer immunity. For example, MSCs can enhance the killing effect of immune cells on tumors by secreting PD-1/PD-L1 antibodies. Furthermore, chimeric antigen receptor (CAR)-redirected T cells have been proven to be efficacious in the treatment of hematologic malignancy; however, CAR-redirected T cells showed less capacity to eliminate solid tumors due to the barrier of the TME. As such, MSCs can be used to generate “tracks” and guide CAR-T cells into the tumor site while continually secreting supporting factors to maintain the activity of CAR-T cells [179].

We expect that these approaches will be promising strategies in the search for efficient and safe nonviral vectors.

## Figures and Tables

**Table 1:** GDEPT of cancers using MSCs.

Factor	Host	Tumor Model	Vectors	MSCs/Tumor Cells	Reference
TK (GCV)	Nude mice	Glioma	Retroviral	1:1	[53]
	Nude mice	Human glioma	Baculoviral	1:1	[54]
	Nude mice	Mice melanoma	Nonviral	1:1 to 1:64	[55]
	Nude mice	Glioblastomas	Lentiviral	4:1	[56]
	Nude mice	Glioma	Adenoviral	1:1	[57]
	Human	Gastrointestinal cancer	Retroviral	—	[52,58,59]
CDy::UPRT (5-FC)	Nude mice	Human colon cancer	Retroviral	1:1	[60]
	Nude mice	Human Prostate cancer	Retroviral	2:3	[61]
	Nude mice	Human melanoma	Retroviral	1:5	[62]
CD (5-FC)	Nude mice	Rat glioma	Adenoviral	1:1	[63]
	Nude mice	Human gastric cancer	Nonviral	1:2	[64]

**Table 2:** Bioactive molecule therapy for cancers using MSCs.

Factor	Mechanism	Tumor Model	Vector	MSCs/Tumor Cells	Reference
IFN-α	Induction of apoptosis	Melanoma	Adenoviral	10:1	[71]
IFN-β	Induction of differentiation	Glioma, prostate cancer	Adenoviral	1:2 or 1:2.5	[24,72]
IFN-γ	Induction of apoptosis	Lung cancer	Lentiviral	1:3.3	[73]
IL-2	Immunomodulation	Glioma	Adenoviral	10:1	[74]
IL7	Th1 polarization	Colorectal cancer	Retroviral	1:6	[75]
IL-12	Activation of T and NK cells	Renal cell carcinoma, glioma	Adenoviral	1:20 or 1:1	[76,77]
IL-15	Activation of T and NK cells	Pancreatic cancer	Lentiviral	1:1	[70]
IL-25	Proapoptosis	Pancreatic cancer	—	—	[78]
CX3CL1	Activation of T and NK cells	melanoma, colon cancer	Adenoviral	1:1	[79]
TRAIL	Induction of apoptosis	Pancreatic cancer	Lentiviral	1:2 or 1:3	[80,81,82]
TSP-1	Antiangiogenesis	Glioblastoma	Lentiviral	1:2.5	[83]
NK4	Induction of apoptosis	Colon cancer	Adenoviral	1:1	[84]

## Data Availability

Not applicable.

## Supplementary Materials

The following are available online at https://www.mdpi.com/article/10.3390/pharmaceutics13060913/s1, Table S1: The efficiency of non-viral strategies for MSC engineering.
