# Supplementary material for: Mesenchymal Stem Cells Engineered by Nonviral Vectors: A Powerful Tool in Cancer Gene Therapy
Source: Pharmaceutics. 2021 Jun 21;13(6):913. doi: 10.3390/pharmaceutics13060913 (PMC8235299; doi:10.3390/pharmaceutics13060913)
Supplement: Supplementary file 1 [file pharmaceutics-13-00913-s001.zip › pharmaceutics-1234488-supplementary.pdf]

pharmaceutics

Mesenchymal Stem Cells Engineered by Nonviral Vectors: A Powerful Tool in Cancer Gene Therapy

Yuan Ding, Chenyang Wang, Zhongquan Sun, Yingsheng Wu, Wanlu You, Zhengwei Mao and Weilin Wang

Table S1. The efficiency of non-viral strategies for MSC engineering.

Factor	Transfection Method	Vector	Efficiency	Cell Death	Reference
TRAIL	Photochemical internalization	Branched PEI	>10 fold	<5%	[1]
CD (5-FC)	Fusogenic lipids	Linear PEI	~80%	<5%	[2]
CXCR4	Microporation electroporation	Minicircles	~66%	4%–13%	[3]
—	VEGFR-1 targeted	Biomimetic vectors	Over 50%	<25%	[4]
GFP	GD2 targeted	PEG-g-PEI-SPIO	~60%	<20%	[5]
—	nAchR targeted	RVG-PAM-ABP	~60%	<5%	[6]
BMP2	3D tissue engineered scaffold	Dextran-plasmid	~7 fold	—	[7]
Ascl1, Brn2, Sox2	—	Carbon dots	>10 fold	<5%	[8]
GFP	pEGFP and pLuc	PEI-modified nanoparticles	~75%	—	[9]
Sox9	Lipofectamine	Minicircle	~40%	—	[10]
siRNA	Opti-MEM	PIC	85%–93%	<20%	[11]
GFP	—	pDNA/PEI/HA	~2 fold	—	[12]
SOX9	pEGFP and pLuc	PLGA	75%	—	[13]

References

1. Han, J.; Hwang, H.S.; Na, K. TRAIL-secreting human mesenchymal stem cells engineered by a non-viral vector and photochemical internalization for pancreatic cancer gene therapy. *Biomaterials* **2018**, *182*, 259–268.
2. Ho, Y.K.; Woo, J.Y.; Tu, G.X.E.; Deng, L.W.; Too, H.P. A highly efficient non-viral process for programming mesenchymal stem cells for gene directed enzyme prodrug cancer therapy. *Sci. Rep.* **2020**, *10*, 14257.
3. Mun, J.Y.; Shin, K.K.; Kwon, O.; Lim, Y.T.; Oh, D.B. Minicircle microporation-based non-viral gene delivery improved the targeting of mesenchymal stem cells to an injury site. *Biomaterials* **2016**, *101*, 310–320.
4. Chen, X.; Nomani, A.; Patel, N.; Nouri, F.S.; Hatefi, A. Bioengineering a non-genotoxic vector for genetic modification of mesenchymal stem cells. *Biomaterials* **2018**, *152*, 1–14.
5. Pang, P.; Wu, C.; Shen, M.; Gong, F.; Zhu, K.; Jiang, Z.; Guan, S.; Shan, H.; Shuai, X. An MRI-visible non-viral vector bearing GD2 single chain antibody for targeted gene delivery to human bone marrow mesenchymal stem cells. *PloS One* **2013**, *8*, e76612.
6. Beloor, J.; Ramakrishna, S.; Nam, K.; Seon Choi, C.; Kim, J.; Kim, S.H.; Cho, H.J.; Shin, H.; Kim, H.; Kim, S.W., et al. Effective gene delivery into human stem cells with a cell-targeting Peptide-modified bioreducible polymer. *Small* **2015**, *11*, 2069–2079.
7. Hosseinkhani, H.; Azzam, T.; Kobayashi, H.; Hiraoka, Y.; Shimokawa, H.; Domb, A.J.; Tabata, Y. Combination of 3D tissue engineered scaffold and non-viral gene carrier enhance in vitro DNA expression of mesenchymal stem cells. *Biomaterials* **2006**, **27**, 4269–4278.
8. Chen, J.; Wang, Q.; Zhou, J.; Deng, W.; Yu, Q.; Cao, X.; Wang, J.; Shao, F.; Li, Y.; Ma, P., et al. Porphyra polysaccharide-derived carbon dots for non-viral co-delivery of different gene combinations and neuronal differentiation of ectodermal mesenchymal stem cells. *Nanoscale* **2017**, *9*, 10820–10831.
9. Park, J.S.; Na, K.; Woo, D.G.; Yang, H.N.; Kim, J.M.; Kim, J.H.; Chung, H.M.; Park, K.H. Non-viral gene delivery of DNA polyplexed with nanoparticles transfected into human mesenchymal stem cells. *Biomaterials* **2010**, *31*, 124–132.

10. Tidd, N.; Michelsen, J.; Hilbert, B.; Quinn, J.C. Minicircle Mediated Gene Delivery to Canine and Equine Mesenchymal Stem Cells. *Int. J. Mol. Sci.* **2017**, *18*, 819.
11. Raisin, S.; Morille, M.; Bony, C.; Noël, D.; Devoisselle, J.M.; Belamie, E. Tripartite polyionic complex (PIC) micelles as non-viral vectors for mesenchymal stem cell siRNA transfection. *Biomater. Sci.* **2017**, *5*, 1910–1921.
12. Cho, S.H.; Noh, Y.W.; Cho, M.Y.; Lim, Y.T. An Electrostatically Self-Assembled Ternary Nanocomplex as a Non-Viral Vector for the Delivery of Plasmid DNA into Human Adipose-Derived Stem Cells. *Molecules* **2016**, *21*, 572.
13. Kim, J.H.; Park, J.S.; Yang, H.N.; Woo, D.G.; Jeon, S.Y.; Do, H.J.; Lim, H.Y.; Kim, J.M.; Park, K.H. The use of biodegradable PLGA nanoparticles to mediate SOX9 gene delivery in human mesenchymal stem cells (hMSCs) and induce chondrogenesis. *Biomaterials* **2011**, *32*, 268–278.
